# The relationship between pain duration characteristics and pain intensity in herpes zoster-related pain: a single-center retrospective study

**DOI:** 10.3389/fmed.2024.1466214

**Published:** 2024-11-07

**Authors:** Liu Wang, Xiaoxiao Lan, Zhixuan Lan, Shengrong Xu, Ruilin He, Zongbin Jiang

**Affiliations:** Department of Pain Medicine, The Second Affliated Hospital of Guangxi Medical University, Nanning, China

**Keywords:** herpes zoster, postherpetic neuralgia, pain duration characteristics, pain intensity, inflammation

## Abstract

**Background:**

The varicella-zoster virus (VZV) can cause herpes zoster (HZ), which may progress to postherpetic neuralgia (PHN), leading to severe inflammatory responses and pain.

**Objective:**

This study investigates the relationship between pain duration characteristics and pain intensity in patients with herpes zoster-related pain, hypothesizing that persistent pain correlates with higher pain intensity compared to intermittent pain.

**Methods:**

A retrospective study was conducted at the Second Affiliated Hospital of Guangxi Medical University, China. Data from patients treated for herpes zoster-related pain between January 2019 and February 2024 were analyzed. Pain intensity was measured using the Numerical Rating Scale (NRS-11), and pain duration was categorized as intermittent or persistent. Multivariate regression models were used to assess the association between pain duration and intensity, adjusting for potential confounders.

**Results:**

A total of 840 patients were included. Persistent pain was significantly associated with higher NRS-11 scores compared to intermittent pain (*β* = 0.71, 95% CI 0.50–0.91, *p* < 0.001). Subgroup analyses showed that persistent pain was associated with higher pain intensity in both acute HZ and PHN patients (HZ: *β* = 0.71, 95% CI 0.45–0.96, *p* < 0.001; PHN: *β* = 0.76, 95% CI 0.40–1.13, *p* < 0.001). Inflammatory markers, such as C-reactive protein (CRP) and white blood cell count, were positively correlated with pain intensity.

**Conclusion:**

Pain duration significantly impacts pain intensity in HZ patients. Considering pain duration is crucial for effective pain management. Further research should explore the mechanisms underlying persistent pain to develop better treatment strategies.

## 1 Introduction

The varicella-zoster virus (VZV) typically spreads through respiratory droplets and direct contact. When reactivated from its latent state in the dorsal root ganglia, VZV can cause herpes zoster (HZ), leading to immune and inflammatory responses around the nerves, resulting in severe pain (1–3). With an aging population, the health burden associated with VZV is expected to increase, posing a significant global health issue (4). It manifests as a painful rash and can lead to chronic pain conditions such as postherpetic neuralgia (PHN), which is defined as dermatomal pain persisting at least 90 days after the appearance of the acute herpes zoster rash (5). Without vaccination, the lifetime risk of HZ is 30% (6). The burden of pain associated with HZ is substantial, affecting not only physical functioning but also psychological well-being and overall quality of life (7–9). The burden of PHN, however, can be even more severe, as it often becomes chronic and refractory to standard treatments, significantly impairing patients’ quality of life, physical functioning, and psychological well-being, and leading to long-term disability and emotional distress (5). Effective management of herpes zoster-related pain is therefore a critical aspect of patient treatment.

The management of pain duration is a crucial component of pain management. Pain intensity and pain duration have been shown to significantly impair quality of life and mental health (10–13). High pain intensity or prolonged pain duration can negatively impact patients’ muscular function (14), and the duration of neuropathic pain may affect the efficacy of pharmacological treatments (15). Baseline pain duration can also influence patient referrals (16). Despite its clinical importance, the relationship between pain duration characteristics and pain intensity in herpes zoster-related pain remains underexplored. Most existing studies focus on the prevalence and risk factors of PHN (17–19), with limited attention to how pain duration affects pain intensity across different stages of HZ.

Understanding the factors that influence pain intensity is crucial for developing targeted and effective pain management strategies. Previous studies have indicated that the relationship between pain intensity and perceived duration is complex, with high-intensity stimuli (high pain) potentially leading to a longer perceived duration (20). Conversely, when the perceived duration of a pain stimulus is misled to be shorter, the perceived pain intensity may decrease (21). In patients with knee osteoarthritis, some studies suggest that intermittent pain is generally associated with lower pain intensity, while persistent pain may correlate with higher pain intensity (22). However, other studies indicate that intermittent pain can be more severe than persistent pain (23). Therefore, larger sample studies are needed to clarify these discrepancies in the literature. Additionally, previous studies have highlighted the need for comprehensive pain assessments that consider not only intensity but also the duration and type of pain (22). Pain duration characteristics are also used in diagnosing chronic pain conditions (24). The pain experience is multifaceted, influenced by factors such as gender, BMI, and comorbidities (22). However, there is a paucity of evidence on the specific impact of pain duration characteristics, such as intermittent versus persistent pain, on pain intensity in HZ and PHN patients. Different diseases and pain management strategies define pain duration characteristics variably, and their impact on pain is not uniformly concluded (25–28).

Given these gaps in the literature, this study aims to investigate the relationship between pain duration characteristics and pain intensity in patients with herpes zoster-related pain. We hypothesize that patients experiencing persistent pain will report higher pain intensity, as measured by the Numerical Rating Scale (NRS-11), compared to those with intermittent pain. This study aims to provide valuable insights into the complexity of herpes zoster-related pain, enhancing the understanding of the mechanisms underlying herpes zoster-related pain, and offering strategies to improve pain management, thereby improving treatment outcomes and quality of life for patients affected by this debilitating condition.

## 2 Methods

### 2.1 Study design and ethical approval

This retrospective study was conducted at the Second Affiliated Hospital of Guangxi Medical University and received approval from the hospital’s Ethics Committee [2024-KY(0510)]. Informed consent was waived due to the retrospective nature of the study. The study adhered to the principles of the Declaration of Helsinki and followed the Strengthening the Reporting of Observational Studies in Epidemiology (STROBE) guidelines (29).

### 2.2 Study population

Patients treated for herpes zoster-related pain at the Pain Department of the Second Affiliated Hospital of Guangxi Medical University between January 2019 and February 2024 were considered eligible. Each patient was included in the analysis only once. Herpes zoster-related pain includes both acute pain associated with HZ and PHN. HZ is characterized by a painful, unilateral vesicular rash in a dermatomal distribution, occurring during the active phase of varicella-zoster virus reactivation. PHN, on the other hand, is defined as dermatomal pain persisting for at least 90 days after the appearance of the acute herpes zoster rash. Exclusion criteria included patients with mental illnesses affecting accurate pain assessment and those with incomplete or missing records.

### 2.3 Data collection

Data were extracted from the hospital’s pain virtual ward system, electronic medical records, hospital information system, and laboratory information system. This comprehensive data source ensured data integrity and traceability, jointly managed by the Pain Department and the Information Department.

### 2.4 Collected variables

#### 2.4.1 Basic patient information

Data collected included body mass index (BMI), gender, age, marital status, smoking history, and alcohol use history.

#### 2.4.2 Pain-related information

Pain location: Specific areas of HZ pain were documented.
Pain intensity: Measured using the NRS-11, where patients rated their pain on a scale from 0 (no pain) to 10 (worst possible pain) (30).
Pain type: Classified using the DN4 questionnaire and the Chinese version of the Neuropathic Pain Diagnostic and Treatment Assessment Scale, encompassing types like burning, pins and needles, electric shock, tingling, itching, and others (tearing pain, throbbing pain, pulling pain, twitching pain, and aching pain) (31, 32).
Pain duration characteristics: Categorized as intermittent or continuous pain, with continuous pain defined as pain persisting throughout the day and intermittent pain having significant relief periods within a day.
Allodynia: Documented as pain due to a stimulus that does not normally provoke pain (33).
Laboratory data: Collected from fasting peripheral venous blood samples, including globulin, albumin, hemoglobin, C-reactive protein (CRP), cystatin C, platelet count, red blood cell count, urea, and white blood cell count.
Charlson comorbidity index (CCI): Used to assess comorbidities such as myocardial infarction, congestive heart failure, cerebrovascular disease, diabetes, moderate/severe renal disease, tumor, leukemia, lymphoma, moderate/severe liver disease, metastatic tumor, and acquired immunodeficiency syndrome (AIDS) (34, 35).

### 2.5 Data usage statement

All data used in this study were sourced from the pain virtual ward system, electronic medical record system, hospital information system, and laboratory information system of the Second Affiliated Hospital of Guangxi Medical University, and were extracted following an information security review and approval. These data will be used for further analyses, including identifying additional risk factors, developing pain prediction models, and analyzing pain trajectories. To ensure academic integrity and data transparency, we declare that subsequent studies may continue to use the same or updated datasets to facilitate comparison and comprehensive analysis of different research outcomes.

### 2.6 Statistical analysis

All analyses were conducted utilizing R Statistical Software (Version 4.2.2, http://www.R-project.org, The R Foundation) and Free Statistics analysis platform (Version 1.8, Beijing, China). Continuous data were summarized as mean ± standard deviation (SD) or median and interquartile range (IQR). Categorical variables were represented as frequencies and percentages. Missing values were noted for several variables, with the majority being below 5%. Given the low proportion of missing data and its occurrence primarily in covariates, no special handling was performed for missing values (36).

For continuous data with normal distribution, independent samples *t*-tests were used for comparisons between two groups; for non-normally distributed continuous data, Mann–Whitney *U* tests were used. Categorical variables were compared using Pearson chi-square tests or Fisher’s exact tests. A *p*-value <0.05 was considered statistically significant, and results were presented as odds ratios (OR) with 95% confidence intervals (95% CI).

Univariate linear regression models were first used to assess the relationship between baseline variables and NRS-11 scores. Variables with *p* < 0.05 in the univariate analysis were considered for inclusion in the multivariate model (Model 2). Model 2 adjusted for age, BMI, smoking status, pain type, albumin, hemoglobin, CRP, cystatin C, and white blood cell count. To ensure a comprehensive adjustment for potential confounders, Model 3 included all variables from Model 2 and additional demographic and clinical factors: gender, marital status, alcohol use, CCI, presence of allodynia, and pain location. Subgroup analyses were conducted to examine the association between pain duration characteristics and NRS-11 scores within specific patient subgroups, including those with HZ and PHN.

## 3 Results

### 3.1 Baseline characteristics

**Figure 1:**
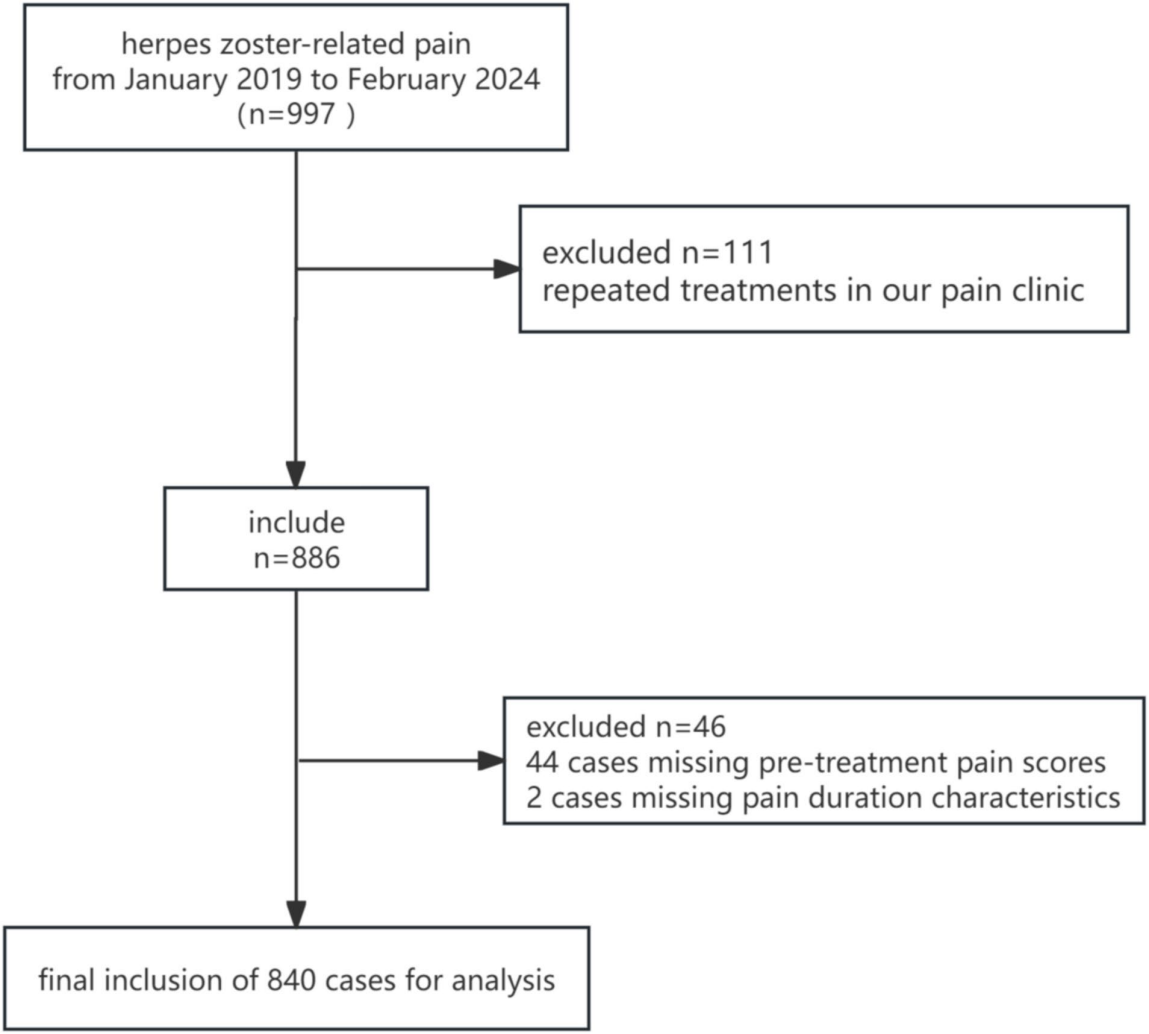
Flowchart of patient inclusion.

Between January 2019 and February 2024, a total of 997 patients with herpes zoster-related pain were enrolled in this study. After excluding 111 cases due to repeated treatments, 44 cases with missing pre-treatment pain scores, and 2 cases with missing pain duration characteristics, 840 patients were included in the final analysis (Figure 1). The baseline characteristics and pain features of the study population, stratified by pain duration characteristics, are summarized below.

The majority of the patients had intermittent pain (*n* = 630), while a smaller proportion had persistent pain (*n* = 210). The overall mean NRS-11 score was 4.9 ± 1.3, with a statistically significant difference observed between the intermittent and persistent pain groups (intermittent: 4.7 ± 1.3, persistent: 5.4 ± 1.3, *p* < 0.001). Most baseline characteristics were similar between the groups, except for BMI, which was significantly different (*p* = 0.002) (Table 1).

**Table 1:** Baseline characteristics of patients with herpes zoster-related pain stratified by pain duration.^a^

Variables	Total (*n* = 840)	Intermittent (*n* = 630)	Persistent (*n* = 210)	*p*	Statistic
NRS-11 score	4.9 ± 1.3	4.7 ± 1.3	5.4 ± 1.3	<0.001	52.064
Pain type				0.094	9.412
Burning	296 (35.2)	233 (37)	63 (30)		
Pins and needles	315 (37.5)	219 (34.8)	96 (45.7)		
Electric shock	62 (7.4)	48 (7.6)	14 (6.7)		
Tingling	43 (5.1)	31 (4.9)	12 (5.7)		
Itching	30 (3.6)	25 (4)	5 (2.4)		
Others^b^	94 (11.2)	74 (11.7)	20 (9.5)		
Allodynia				0.684	0.166
No	334 (39.8)	248 (39.4)	86 (41)		
Yes	506 (60.2)	382 (60.6)	124 (59)		
PHN				0.166	1.923
No	611 (72.7)	466 (74)	145 (69)		
Yes	229 (27.3)	164 (26)	65 (31)		
Gender				0.905	0.014
Male	431 (51.3)	324 (51.4)	107 (51)		
Female	409 (48.7)	306 (48.6)	103 (49)		
Age	66.5 ± 11.9	66.5 ± 11.8	66.7 ± 12.2	0.877	0.024
Marital status				0.325	0.969
Single^c^	100 (11.9)	79 (12.5)	21 (10)		
Married	740 (88.1)	551 (87.5)	189 (90)		
BMI	22.7 ± 3.3	22.9 ± 3.2	22.1 ± 3.4	0.002	9.776
Smoking				0.407	0.687
No	742 (89.5)	559 (90)	183 (88)		
Yes	87 (10.5)	62 (10)	25 (12)		
Alcohol use				0.951	0.004
No	770 (92.9)	577 (92.9)	193 (92.8)		
Yes	59 (7.1)	44 (7.1)	15 (7.2)		
Left or right side				0.155	2.027
Left	425 (51.1)	329 (52.6)	96 (46.8)		
Right	406 (48.9)	297 (47.4)	109 (53.2)		
Location				0.108	6.071
Head and face	193 (23.0)	141 (22.4)	52 (24.8)		
Neck and upper limbs	142 (16.9)	116 (18.4)	26 (12.4)		
Chest and abdomen	414 (49.3)	311 (49.4)	103 (49)		
Lower back and legs	91 (10.8)	62 (9.8)	29 (13.8)		
CCI	0.0 (0.0, 1.0)	0.0 (0.0, 1.0)	0.0 (0.0, 1.0)	0.779	0.079
Globulin	26.1 ± 4.7	26.2 ± 4.7	25.7 ± 4.5	0.152	2.054
Albumin	38.0 ± 3.8	38.2 ± 3.8	37.6 ± 3.9	0.06	3.542
Hemoglobin	120.0 ± 23.7	120.7 ± 22.3	117.9 ± 27.5	0.15	2.072
CRP	3.3 (1.8, 7.9)	3.3 (1.7, 7.8)	3.4 (1.9, 7.9)	0.529	0.396
Cystatin C	1.2 ± 0.5	1.2 ± 0.6	1.2 ± 0.3	0.138	2.202
Platelet count	242.1 ± 81.9	239.8 ± 79.9	249.0 ± 87.6	0.162	1.96
Red blood cell count	4.2 ± 0.6	4.2 ± 0.7	4.2 ± 0.6	0.290	1.121
Urea	5.3 ± 2.4	5.4 ± 2.5	5.1 ± 2.0	0.095	2.801
White blood cell count	6.7 ± 2.5	6.7 ± 2.6	6.7 ± 2.2	0.85	0.036

a Missing values were noted for several variables, including alcohol use (1.31%), BMI (3.45%), left or right (1.07%), smoking (1.31%), globulin (2.02%), red blood cell count (1.67%), urea (2.26%), white blood cell count (1.67%), albumin (2.02%), hemoglobin (1.67%), CRP (4.64%), cystatin C (2.26%), and platelet count (1.67%). Data are expressed as the mean ± SD, median (interquartile range), or percentage.

b Including tearing pain, throbbing pain, pulling pain, twitching pain, and aching pain.

c Including unmarried, divorced, widowed.

PHN, postherpetic neuralgia; CCI, Charlson comorbidity index; BMI, body mass index; CRP, C-reactive protein.

### 3.2 Correlation analysis

Correlation analysis (Table 2) indicated a significant relationship between pain duration characteristics and NRS-11 scores. In the unadjusted model, patients with persistent pain had significantly higher NRS-11 scores compared to those with intermittent pain (*β* = 0.74, 95% CI 0.54–0.94, *p* < 0.0001). Additionally, variables such as the type of pain (pins and needles), age, smoking status, BMI, albumin, hemoglobin, CRP, cystatin C, and white blood cell count were significantly correlated with NRS-11 scores (*p* < 0.05).

**Table 2:** The unadjusted association between baseline variables and NRS-11 score.

Variables	Coeff. (95% CI)	*p*
Pain duration characteristics		
Intermittent	Reference	
Persistent	0.74 (0.54, 0.94)	<0.0001
Pain type		
Burning	Reference	
Pins and needles	0.26 (0.05, 0.47)	0.014
Electric shock	0.07 (−0.29, 0.44)	0.686
Tingling	0.01 (−0.41, 0.43)	0.962
Itching	−0.05 (−0.54, 0.45)	0.852
Others^a^	−0.12 (−0.43, 0.19)	0.439
Allodynia		
No	Reference	
Yes	0.11 (−0.08, 0.29)	0.2484
PHN		
No	Reference	
Yes	0.02 (−0.18, 0.22)	0.8737
Gender		
Male	Reference	
Female	−0.14 (−0.32, 0.04)	0.1183
Age	0.02 (0.01, 0.03)	<0.0001
Marital status		
Single^b^	Reference	
Married	0.27 (−0.00, 0.55)	0.0528
BMI	−0.03 (−0.06, −0.00)	0.0345
Smoking		
No	Reference	
Yes	0.36 (0.07, 0.65)	0.0165
Alcohol use		
No	Reference	
Yes	0.03 (−0.32, 0.38)	0.8861
Left or right side		
Left	Reference	
Right	−0.10 (−0.28, 0.07)	0.2536
Location		
Head and face	Reference	
Neck and upper limbs	−0.13 (−0.41, 0.16)	0.3865
Chest and abdomen	−0.11 (−0.33, 0.12)	0.3472
Lower back and legs	0.21 (−0.12, 0.54)	0.2124
CCI	−0.00 (−0.07, 0.06)	0.977
Globulin	0.00 (−0.02, 0.02)	0.8283
Albumin	−0.05 (−0.07, −0.03)	<0.0001
Hemoglobin	−0.00 (−0.01, −0.00)	0.0149
CRP	0.01 (0.00, 0.01)	<0.0001
Cystatin C	0.18 (0.01, 0.35)	0.0403
Platelet count	0.00 (−0.00, 0.00)	0.2157
Red blood cell count	−0.10 (−0.24, 0.03)	0.1405
Urea	−0.00 (−0.04, 0.04)	0.9767
White blood cell count	0.05 (0.02, 0.09)	0.0033

PHN, postherpetic neuralgia; CCI, Charlson comorbidity index; BMI, body mass index; CRP, C-reactive protein. a: Including tearing pain, throbbing pain, pulling pain, twitching pain, and aching pain. b: Including unmarried, divorced, widowed.

### 3.3 Multivariate regression analysis

Multivariate regression analysis (Table 3) demonstrated a significant association between pain duration characteristics and NRS-11 scores. In the unadjusted model, persistent pain was significantly associated with higher NRS-11 scores (*β* = 0.74, 95% CI 0.54–0.94, *p* < 0.001). After adjusting for age, BMI, smoking status, pain type, albumin, hemoglobin, CRP, cystatin C, and white blood cell count, persistent pain remained significantly associated with higher NRS-11 scores (*β* = 0.72, 95% CI 0.51–0.92, *p* < 0.001). Further adjustment for gender, marital status, alcohol use, and CCI did not alter the significance of the association (*β* = 0.71, 95% CI 0.51–0.91, *p* < 0.001).

**Table 3:** Multivariate Regression Analysis of NRS-11 Scores.

Pain duration characteristics	*n*	Model 1 crude *p*-value		Model 2		Model 3	
		*β*-coefficients (95% CI)	*p*-value	*β*-coefficients (95% CI)	*p*-value	*β*-coefficients (95% CI)	*p*-value
Intermittent	630	0 (Ref)		0 (Ref)		0 (Ref)	
Persistent	210	0.74 (0.54–0.94)	<0.001	0.72 (0.51–0.92)	<0.001	0.71 (0.50–0.91)	<0.001

Model 1: Unadjusted covariates.

Model 2: Adjusted for age, BMI, smoking, pain type, albumin, hemoglobin, CRP, cystatin C, and white blood cell count.

Model 3: Adjusted for Model 2 + gender, marital status, alcohol use, CCI, allodynia, and location.

### 3.4 Subgroup analysis

Subgroup analysis (Table 4) results are summarized as follows: persistent pain was significantly associated with higher NRS-11 scores in both HZ and PHN patients. In HZ patients, persistent pain was associated with significantly higher NRS-11 scores compared to intermittent pain (adjusted Model 3: *β* = 0.71, 95% CI 0.45–0.96, *p* < 0.001). Similarly, in PHN patients, persistent pain was associated with significantly higher NRS-11 scores (adjusted Model 3: *β* = 0.76, 95% CI 0.40–1.13, *p* < 0.001).

**Table 4:** Subgroup analysis of NRS-11 scores by pain duration.

Subgroup	Pain duration characteristics	*n*	Model 1		Model 2		Model 3	
			*β*-coefficients (95% CI)	*p*-value	*β*-coefficients (95% CI)	*p*-value	*β*-coefficients (95% CI)	*p*-value
Herpes Zoster	Intermittent	466	0 (Ref)		0 (Ref)		0 (Ref)	
	Persistent	145	0.73 (0.48–0.97)	<0.001	0.74 (0.48–0.99)	<0.001	0.71 (0.45–0.96)	<0.001
PHN	Intermittent	164	0 (Ref)		0 (Ref)		0 (Ref)	
	Persistent	65	0.77 (0.42–1.11)	<0.001	0.73 (0.38–1.09)	<0.001	0.76 (0.40–1.13)	<0.001

Model 1: Unadjusted.

Model 2: Adjusted for age, BMI, smoking, pain type, albumin, hemoglobin, CRP, cystatin C, and white blood cell count.

Model 3: Adjusted for Model 2 + gender, marital status, alcohol use, CCI, allodynia, and location.

## 4 Discussion

This single-center retrospective study systematically explored the relationship between pain duration characteristics and pain intensity in patients with herpes zoster-related pain. Different diseases and pain management strategies necessitate varying definitions of pain duration (25–28). Given the typically short disease course in most HZ patients, with some seeking treatment on the first day of pain, we defined continuous pain as pain that persists throughout the entire day regardless of its intensity, and intermittent pain as pain that includes significant periods of relief within the same day. Our multivariate regression analysis revealed that persistent pain is significantly associated with higher NRS-11 scores, even after adjusting for various demographic and clinical factors such as age, BMI, smoking status, pain type, albumin, hemoglobin, CRP, cystatin C, white blood cell count, gender, marital status, alcohol use, CCI, presence of allodynia, and pain location (*β* = 0.71, 95% CI 0.51–0.92, *p* < 0.001). This finding underscores the critical role of pain duration in influencing pain intensity.

Our study is the first to investigate the relationship between pain duration characteristics and pain intensity in a large sample size of HZ patients. The results indicate that intermittent pain (*n* = 630, 75%) is the predominant type, while persistent pain *n* = 210, (25%) accounts for only a quarter of the study population. Despite the smaller proportion, patients with persistent pain had significantly higher NRS-11 scores compared to those with intermittent pain, highlighting the critical role of pain duration in influencing pain intensity. Subgroup analysis further revealed that persistent pain was significantly associated with higher NRS-11 scores in both acute HZ (characterized by pain associated with a vesicular rash) and PHN (dermatomal pain persisting for at least 90 days) patients (adjusted Model 3: HZ: *β* = 0.71, 95% CI 0.45–0.96, *p* < 0.001; PHN: *β* = 0.76, 95% CI 0.40–1.13, *p* < 0.001). These findings underscore the importance of considering pain duration in the management of herpes zoster-related pain across different stages.

Pain intensity is not merely a single instantaneous measure but a complex phenomenon closely linked to the duration of pain (37). The duration of pain is also associated with quality of life and psychological health (38). Previous studies have shown that common symptoms in chronic pain conditions, such as pain intensity and symptoms of depression and anxiety, carry important information for identifying clinically relevant subgroups (39). Our findings suggest that the state of pain on the day of hospital admission is related to pain severity, and the duration of pain should be given high priority in the management of herpes zoster-related pain at different stages.

Perceived pain stimulus duration may affect perceived pain intensity, with shorter perceived durations leading to reduced perceived intensity (21). Previous research has shown that pro-inflammatory cytokines, which are part of the immune response to the varicella-zoster virus, are associated with pain intensity in chronic pain patients (40). Although our study was based on patients’ self-reported pain over a single day, we observed positive correlations between white blood cell count and CRP with pain intensity in univariate analysis, suggesting that persistent pain may represent more severe neuropathic changes, such as nerve fiber damage and regeneration, and inflammatory damage (41). These changes can lead to hyperalgesia and continuous nociceptive input, thereby increasing pain intensity (42).

Additionally, one study found a higher incidence of intermittent pain compared to persistent pain in chronic knee arthritis (43). Our study similarly found that in herpes zoster-related pain, both HZ and PHN, intermittent pain (*n* = 630, 75%) patients were nearly three times more common than persistent pain (*n* = 210, 25%) patients. Despite the lower number of patients with persistent pain, their pain intensity was higher. Another study demonstrated that persistent pain has a greater impact on physical activity, indicating the need to consider pain duration as a key feature for accurately predicting pain intensity and formulating appropriate treatment strategies (23).

Our findings suggest that comprehensive management strategies should be adopted for herpes zoster-related persistent pain, but not be limited to antiviral treatments, immunomodulatory therapies, and anti-inflammatory agents. Previous studies have shown that persistent pain is associated with limitations in daily activities, higher comorbidity rates, and depressive symptoms (44). For patients with persistent pain, long-acting formulations may be needed to achieve more stable and prolonged pain control, or the use of the HZ vaccine in high-risk populations (45, 46). Additionally, integrating non-pharmacological treatments, such as physical therapy and psychological interventions, may help alleviate persistent pain (47–49). For intermittent pain patients, short-acting medications before pain onset may reduce the frequency and intensity of pain episodes, and educating patients on the proper use of analgesics can minimize side effects (50, 51).

Our study has several strengths. Firstly, the large sample size provides high statistical power. Secondly, multivariate regression analysis was used to minimize the impact of confounding factors, enhancing the reliability of the results. Thirdly, subgroup analysis further validated the association between persistent pain and higher NRS-11 scores in both HZ and PHN patients, indicating the robustness of this association.

### 4.1 Limitations

As a retrospective study, there are inherent limitations in inferring causality. Data collection may have biases, especially with self-reported pain scores influenced by subjective factors (52). Additionally, the pain intensity is related to various physiological and psychological factors. One limitation of this study is the lack of consideration for psychological factors, such as anxiety, depression, and stress, which are known to significantly influence pain perception. Although collecting detailed psychological data from retrospective records is challenging, future studies should explore the interaction between psychological factors and herpes zoster-related pain to better understand their impact on pain intensity and duration. As a single-center study, the generalizability of our results may be limited. Future research should adopt a prospective design to reduce biases associated with retrospective data collection. Exploring more objective tools, such as quantitative sensory testing or biomarkers, to complement or replace self-reported pain scores could enhance the accuracy and consistency of pain assessments. Studies should also include multi-center data and explore the potential impact of antiviral treatments, vaccination, and other factors on pain duration and intensity.

## 5 Conclusion

Our study systematically reveals a significant association between pain duration and pain intensity in patients with herpes zoster-related pain. The importance of pain duration should be emphasized in clinical assessments and treatment plans. Future studies should further explore the neuropathological and psychological mechanisms underlying persistent pain and develop targeted interventions to improve pain management and patient outcomes. Early identification and intervention for persistent pain patients can help prevent chronic pain and related complications, reducing the progression of PHN and other chronic pain conditions.

## Data Availability

The data analyzed in this study is subject to the following licenses/restrictions: the datasets used in this study are subject to restrictions due to privacy and confidentiality concerns. They are not publicly available and can only be accessed upon reasonable request to the corresponding author, in accordance with the ethical guidelines and data protection regulations of the Second Affiliated Hospital of Guangxi Medical University, and with the approval of the Ethics Committee. Requests to access these datasets should be directed to ZJ, jiangzongbin@sr.gxmu.edu.cn.
